# Does Acupuncture Needling Induce Analgesic Effects Comparable to Diffuse Noxious Inhibitory Controls?

**DOI:** 10.1155/2012/785613

**Published:** 2011-07-05

**Authors:** Juerg Schliessbach, Eveline van der Klift, Andreas Siegenthaler, Lars Arendt-Nielsen, Michele Curatolo, Konrad Streitberger

**Affiliations:** ^1^University Department of Anesthesiology and Pain Therapy, Inselspital Bern, 3010 Bern, Switzerland; ^2^Faculty of Medical Sciences, Radboud University Nijmegen, 6500 HB Nijmegen, Nijmegen, The Netherlands; ^3^Experimental Pain Research Laboratory, Centre for Sensory Motor Interaction, University of Aalborg, 9220 Aalborg, Denmark

## Abstract

Diffuse noxious inhibitory control (DNIC) is described as one possible mechanism of acupuncture analgesia. This study investigated the analgesic effect of acupuncture without stimulation compared to nonpenetrating sham acupuncture (NPSA) and cold-pressor-induced DNIC. Forty-five subjects received each of the three interventions in a randomized order. The analgesic effect was measured using pressure algometry at the second toe before and after each of the interventions. Pressure pain detection threshold (PPDT) rose from 299 kPa (SD 112 kPa) to 364 kPa (SD 144), 353 kPa (SD 135), and 467 kPa (SD 168) after acupuncture, NPSA, and DNIC test, respectively. There was no statistically significant difference between acupuncture and NPSA at any time, but a significantly higher increase of PPDT in the DNIC test compared to acupuncture and NPSA. PPDT decreased after the DNIC test, whereas it remained stable after acupuncture and NPSA. Acupuncture needling at low pain stimulus intensity showed a small analgesic effect which did not significantly differ from placebo response and was significantly less than a DNIC-like effect of a painful noninvasive stimulus.

## 1. Introduction

Acupuncture is frequently used in pain therapy and has a clinically relevant effect on several types of chronic pain disorders [1–3]. However, the mechanism of action of acupuncture remains unclear, and several theories are currently being discussed. Involvement of the endogenous opioid system [4, 5] as well as spinal or supraspinal mechanisms (e.g., gate-control [6, 7], long-term depression [8, 9] or diffuse noxious inhibitory controls [10, 11]) are thought to account for acupuncture-induced pain relief.

The concept of diffuse noxious inhibitory controls (DNIC) has attracted particular attention in the past years. Under normal conditions, pain after application of an experimental nociceptive stimulus is attenuated by a conditioning noxious stimulus to a remote body region [12]. According to experimental animal studies, this effect could contribute to acupuncture analgesia [10, 11]. However, animal experimental settings are different from clinical routine in humans regarding the intensity of acupuncture stimuli [13]. The analgesic effect might therefore be different in animals and humans. 

There are several further considerations that question the involvement of DNIC in acupuncture. Firstly, patients suffering from diseases with typically impaired DNIC (e.g., fibromyalgia [14], osteoarthritis of the hip [15], irritable bowel syndrome [16], or temporomandibular disorder [17]) still benefit from acupuncture therapy [18–21]. Secondly, classic DNIC-inducing tests in humans, such as the cold pressor test, ischemic tourniquet test and thermal heat test are noninvasive but very painful, whereas acupuncture is invasive but usually not very painful. 

To date, several trials in humans have demonstrated an increase in pain thresholds after acupuncture [22, 23] or a decrease in pain ratings [24], but this has, to our knowledge, never been compared to a classical DNIC-inducing test paradigm and to placebo. 

The aim of the present study was to compare the analgesic effects of acupuncture needling without stimulation and cold-pressor-induced DNIC, using nonpenetrating sham acupuncture (NPSA) as a control condition.

## 2. Materials and Methods

### 2.1. Design

This was a randomized, blinded, crossover study. All subjects underwent three interventions on the same day: acupuncture needling, NPSA and cold pressor test. The three tests were performed in a randomized order according to a computer-generated randomization list. The interstimulus interval was set at 10 minutes. In the light of only short duration of stimuli, we considered that wash-out period sufficient.

The study was approved by the local ethics committee and was carried out at the Department of Anesthesiology and Pain Therapy of the University Hospital of Bern. Written informed consent was obtained from all participants.

### 2.2. Volunteers

Forty-five healthy pain-free volunteers were recruited from hospital staff, among medical students and by word of mouth. Minimal age of 18 years and no previous acupuncture experience were required. Exclusion criteria were any ongoing pain, current intake of analgesic drugs, antidepressants or anticonvulsants, vascular disorders of the hand or the foot to be tested, any neurological disorders, diabetes mellitus, alcohol or drug abuse, and pregnancy.

### 2.3. Acupuncture and Nonpenetrating Sham Acupuncture (NPSA)

Acupuncture and NPSA were performed at LI4 (large intestine 4, Hegu), an acupoint which is commonly used for acupuncture analgesia [25]. LI4 is situated between thumb and index finger at the highest point of the first dorsal interosseus muscle. For acupuncture, a 0.3 × 30 mm needle (asia-med, Suhl, Germany) was used. For NPSA, the so-called Streitberger needle [26] (asia-med, Suhl, Germany) was used, which has been evaluated in several studies [27, 28]. The blunt needle tip does not penetrate the skin but instead moves back inside its shaft when slight pressure is applied. When the needle tip touches the skin, the subject feels a pricking sensation. The subjects were told that two different types of needles were used but were unaware of one of them being a non-penetrating needle.

Subjects were blinded to the acupuncture and NPSA needles by placing a plastic ring over the acupuncture point and fixing it with a plaster (Durapore Surgical Tape, 3 M, Minn, USA). The needles were placed through the plaster in the middle of the plastic ring which served as a holder for the nonpenetrating needle. Blinding of the acupuncturist was not possible. The needles were left in place for 5 minutes without further stimulation and the subjects rated the perceived pain intensity on a 0–10 numeric rating scale (NRS) immediately after needle removal, with 0 = no pain and 10 = worst pain imaginable.

### 2.4. Assessment of DNIC

Volunteers immersed their hand in ice-saturated water (1.5 ± 1°C) for a maximum of two minutes. The water was constantly recirculated in order to avoid laminar warming around the hand. If the pain was considered intolerable before 2 minutes had elapsed, subjects withdrew their hand and the elapsed time was noted. Maximal perceived pain intensity was rated on a 0–10 NRS immediately after hand withdrawal.

### 2.5. Pressure Algometry

**Figure 1:**
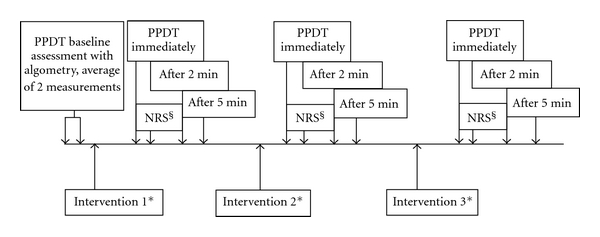
Time line of the experiment. PPDT: pressure pain detection threshold, NRS: numeric rating scale. *Acupuncture, nonpenetrating sham acupuncture (NPSA), and DNIC test were given in randomized order. The duration of acupuncture and NPSA was 5 minutes, and the maximal duration of the DNIC test was 2 minutes. ^§^The subjects were asked to rate the pain intensity during the intervention on a 0–10 NRS (0 = no pain, 10 = worst pain imaginable).

Pressure pain detection threshold (PPDT) at the ipsilateral second toe was measured by a second investigator who was blinded by a curtain drawn across the patient so that he could not see whether acupuncture or NPSA was in progress. An electronic algometer (Somedic AB, Horby, Sweden) with a probe area of 1 cm^2^ was used. Pressure was increased from 0 to a maximum of 1000 kPa at a rate of 30 kPa/s. The subjects were instructed to stop the measuring at the moment when the pressure sensation turned to pain (detection threshold) by pressing the button. First, several measurements were performed on the opposite foot for training purposes. Then two assessments of PPDT were made at the second toe, the mean of which represented the baseline threshold. Subsequent assessments of PPDT were made after each of the three interventions immediately after removal of the acupuncture needles or hand withdrawal from the ice water (time 0) and after 2 and 5 minutes.  Figure 1 depicts the time flow of the experiments.

### 2.6. Data Analysis

Assuming that NPSA does not induce a considerable analgesic effect, sample size was calculated based on the difference between acupuncture and NPSA at time 0. Approximately 8% PPDT elevation after acupuncture at LI4 was found in the study of Zaslawski et al. [23] Expecting a similar effect in our population and assuming a standard deviation of 18%, examination of 43 subjects would provide 80% power. We tested 45 subjects in order to account for possible higher variability.

Based on the two measurements before the interventions, the arithmetical mean was calculated and considered the baseline pressure pain threshold. This baseline value was subtracted from each of the subsequently obtained measurements and a two-way repeated measures ANOVA was performed on these differences. Differences to baseline instead of absolute values were chosen for easier depiction. The two factors investigated were (i) intervention with the three levels “acupuncture”, “NPSA” and “DNIC test”, and (ii) time with the levels “0 minutes”, “2 minutes” and “5 minutes.”

## 3. Results

Forty-five healthy volunteers were enrolled (23 females, 22 males) with a mean age of 24.2 years (SD 5.7). Female subjects were aged 24.6 years (SD 7.5), while male subjects were aged 23.9 (SD 3.0). There was no significant age difference between males and females (*P* = 0.67). PPDT at baseline was 299 kPa (SD 112) and rose to 364 kPa (SD 144), 353 kPa (SD 135), and 467 kPa (SD 168) immediately after acupuncture, NPSA, and DNIC test, respectively. The absolute values for PPDT assessments at every time point are presented in Table 1. PPDT after each of the three interventions was significantly different from the baseline threshold at all time points (*P* < 0.001).

**Figure 2:**
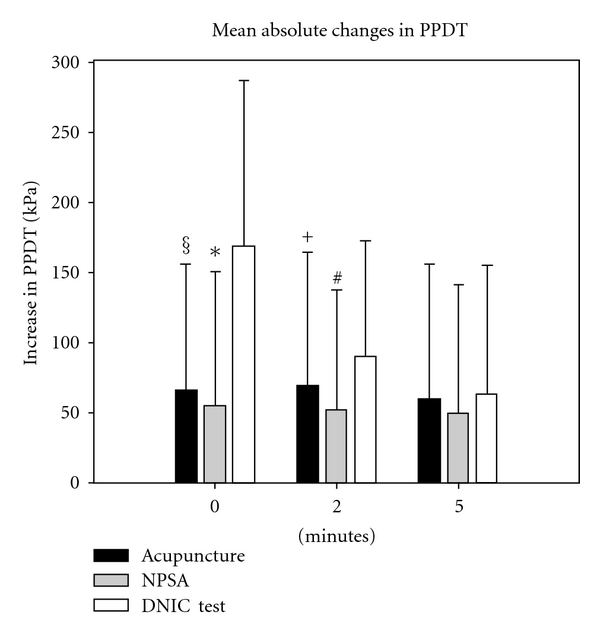
Comparison of pain threshold elevation at the end of acupuncture, NPSA, and DNIC test. PPDT: pressure pain detection threshold, NPSA: nonpenetrating sham acupuncture, DNIC: diffuse noxious inhibitory control. Acupuncture and NPSA are significantly different from DNIC test at 0 and 2 minutes. ^§^ *P* < 0.001, *P < 0.001, ^+^ *P* = 0.05, ^#^ *P* < 0.001.

Figure 2 displays the absolute changes in PPDT during the five-minute posttest period after each of the interventions. We found a significantly higher PPDT-increase in the DNIC test compared to acupuncture, and no significant difference between acupuncture and NPSA. Comparison between the three interventions using the Student-Newman-Keuls method for all-pairwise comparisons showed statistically significant differences between DNIC test and acupuncture at 0 and at 2 minutes (*P* < 0.001 and *P* = 0.05, resp.) as well as between DNIC test and NPSA at 0 and at 2 minutes (both *P* < 0.001). There was no statistically significant difference between any of the interventions after 5 minutes. 

There was a trend for greater PPDT changes after acupuncture than after NPSA throughout all measurements, but the difference was not significant at any of the time points (*P* = 0.353 at 0 minutes, *P* = 0.15 at 2 minutes, and *P* = 0.399 at 5 minutes). 

The measurements after DNIC test displayed a significant decrease over time, with *P* < 0.001 for both 0 versus 2 and 0 versus 5 minutes and *P* = 0.013 for 2 versus 5 minutes. There was no time dependency for PPDT change after both acupuncture and NPSA.

The pain intensity during each of the three different procedures was rated on a 0–10 NRS. Verum acupuncture was rated as 2.4 (SD 1.5), NPSA was rated as 1.1 (SD 0.9), and ice water was rated as 7.1 (SD 1.5). These differences were statistically significant (*P* < 0.001).

## 4. Discussion

The present study showed a similar increase in pressure pain thresholds after acupuncture and NPSA, but neither the magnitude nor the time profile was similar to the DNIC effect evoked by the more painful cold-pressor test. Unlike the classic DNIC response, which was short lasting and decreasing over time, acupuncture produced a constant and only moderate pain threshold elevation. Moreover, our study showed no significant difference in analgesic effect between acupuncture and NPSA in healthy, pain-free subjects, although there was a trend for greater increase in PPDT after acupuncture than after NPSA. This remains to be verified in a patient population, since healthy volunteers possibly react differently to acupuncture treatment than pain patients do: studies by Napadow et al. have shown that patients with carpal tunnel syndrome responded to acupuncture with more pronounced fMRI signal increase in the hypothalamus and signal decrease in the amygdala compared to healthy controls [29, 30]. In a pain patient population with possibly altered DNIC, a difference between verum acupuncture and NPSA might be more pronounced. 

It has been shown in animal studies that acupuncture activates neuronal pathways which are involved in DNIC [31]. However, findings of animal studies seem not to be transposable to human acupuncture since the experimental settings largely differ from clinical routine-acupuncture [32], especially in terms of pain intensity [13]: DNIC-like effects in animals were observed at high pain intensities, whereas acupuncture in humans is usually performed with low pain intensities which might be insufficient to induce DNIC. The subjects in the present study received only a single needle without stimulation, whereas in animal studies, usually several needles are simultaneously applied with electrical stimulation. 

A study by Treister et al. [33] has shown that the amount of endogenous analgesia after either noxious or innocuous conditioning stimuli (water immersion at 12°C and 25°C, resp.) is highly correlated to the NRS reported for the corresponding stimulus. Consequently, the average NRS of 2.4 ± 1.5 experienced by our subjects might have been too low to induce a DNIC-like effect as observed after the DNIC test (NRS 7.5 ± 1.5). Stronger stimulation of the needle by manual or electrical stimulation might increase the NRS and evoke a DNIC-like effect, as shown in a study by Barlas et al. [34]: they observed a significantly higher PPDT after acupuncture with strong electrical stimulation than after acupuncture with weak stimulation or NPSA. Strong electrical stimulation was defined as “to tolerance, but subnoxious,” whereas weak stimulation was “strong but comfortable.” The weak stimulation resulted in similar PPDT values as NPSA. Conceivably, acupuncture needles can be intensely stimulated, until the pain is strong enough to induce a DNIC response, but under therapeutic conditions (i.e., no or weak stimulation, low NRS), relevant contribution of DNIC to acupuncture analgesia is questionable. Hence our findings do not imply that acupuncture and DNIC are mechanistically different, but they suggest that results from experimental animal studies may not necessarily apply to clinical acupuncture therapy in humans.

Apart from the intensity of the conditioning stimulus, the time profile is another important characteristic of a DNIC response: it is most intense during application of the conditioning stimulus [35] and usually decreases to baseline within 5–10 minutes [36–38]. Although our results show an increase in PPDT after acupuncture, there is no observable decrease during the following 5 minutes, as would be expected if it evoked a DNIC effect.

Possible carry-over effects between treatments represent the major limitation to this study. Although the treatment order was randomized and the interstimulus interval was twice as long as the duration of acupuncture, carry-over effects cannot be completely ruled out. This might certainly be an issue to be addressed in the future.

## 5. Conclusions

Acupuncture at low pain stimulus intensity did not produce a DNIC-like effect comparable to a classical, painful DNIC test and its effect did not significantly differ from the one induced by NPSA. Our results showed that the penetration of an acupuncture needle by itself, though noxious, seems not to induce an analgesic effect mainly mediated by DNIC.

##  Conflict of Interests

The authors have no conflict of interests to declare.

## Figures and Tables

**Table 1:** Absolute values of pressure pain detection thresholds after each of the interventions during the five-minute observation interval. All values are presented as mean (SD). NPSA: nonpenetrating sham acupuncture, DNIC: diffuse noxious inhibitory control.

	baseline	0 minutes	2 minutes	5 minutes
Acupuncture	298.9 (111.7)	364.1 (143.7)	367.4 (143.4)	357.9 (135.9)
NPSA	298.9 (111.7)	353.2 (135.3)	350.6 (133.4)	349.9 (135.6)
DNIC test	298.9 (111.7)	467.0 (169.7)	388.6 (147.4)	361.2 (142.2)
